# Information Needs of Culturally and Linguistically Diverse Women with Endometriosis in Australia: A Qualitative Study

**DOI:** 10.3390/ijerph23030348

**Published:** 2026-03-10

**Authors:** Deniz Senyel, James H. Boyd, Melissa Graham

**Affiliations:** Department of Public Health, School of Psychology and Public Health, La Trobe University, Melbourne 3086, Australia

**Keywords:** women’s health, consumer health information, health communication, health promotion, endometriosis

## Abstract

**Highlights:**

**Public health relevance—How does this work relate to a public health issue?**

- Endometriosis is one of the most common gynaecological disorders, affecting approximately one in seven women in Australia.
- Endometriosis has substantial personal, societal, and economic impacts.

**Public health significance—Why is this work of significance to public health?**

- Accessible and tailored health information is essential for informed decision-making and patient empowerment.
- Understanding the information needs and preferences of CALD women is necessary to provide adequate informational support.

**Public health implications—What are the key implications or messages for practitioners, policy makers and/or researchers in public health?**

- Health education should focus on disease pathogenesis, including the causes, symptoms, and progression of endometriosis, as well as disease management.
- Information resources need to be easy to understand, with minimal text and added visual elements, and should include examples of lived experience with endometriosis. Webinars on endometriosis were requested as an interactive format that allows women to ask questions.

**Abstract:**

Culturally and linguistically diverse (CALD) women make up an important population of women with endometriosis in Australia. However, their experiences with the condition, particularly regarding their experiences with information on endometriosis, have not yet been studied. This qualitative descriptive study aimed to explore the information needs and preferences of CALD women living with endometriosis. A qualitative descriptive study, using semi-structured individual interviews with 11 CALD women aged 23–46 from Victoria, Australia, was undertaken. The data were analysed using thematic analysis. The women’s information needs focused on disease pathogenesis—including causes, symptoms, and progression—as well as disease management, such as medication and surgery, lifestyle factors, fertility treatment, and mental health support. The women suggested resources should be easy to understand, written in simple English, with minimal text and added visual elements. Stories from other women with endometriosis were also recommended, as were interactive formats that enable women to ask questions. While the women primarily preferred written information, they also welcomed formats such as webinars or workshops. This study highlights a need for improved information for CALD women with endometriosis. Information resources should be designed to meet women’s needs in both content and delivery.

## 1. Introduction

Australia is one of the most culturally and linguistically diverse (CALD) countries globally, with 28% of its population born overseas and 23% primarily speaking a language other than English at home [1]. While this diversity enriches Australian society, belonging to a CALD community can create barriers to accessing healthcare, including difficulties navigating the health system, as well as language and cultural challenges [2,3,4]. These barriers are frequently compounded by lower levels of health literacy among CALD populations compared with Australian-born people [3,5]. Lower health literacy is associated with reduced engagement in preventative healthcare, limited control over health-related decisions, and poorer health outcomes [6,7,8,9]. Consequently, individuals with lower health literacy may be less likely to participate in important self-management tasks that are required to enhance health outcomes and manage chronic diseases [10,11]. From a public health perspective, the provision of accessible health information that individuals can understand and apply is crucial, as it subsequently influences healthcare usage and therefore disease prevention and health promotion at a population level [12]. Since health literacy and the usage of health information are further influenced by societal and environmental determinants, understanding CALD communities’ information needs is crucial to improve health outcomes [2,12]. Such interventions should encompass culturally safe healthcare services and informational resources that are responsive to diverse language and literacy needs [2,13,14,15].

Endometriosis is a prevalent and debilitating chronic condition, affecting an estimated one in seven women of reproductive age in Australia [16]. Endometriosis is characterised by the growth of endometrial-like tissue outside the uterus [17]. While these lesions are most commonly located within the pelvic cavity, they can occur elsewhere in the body [18]. Consequently, endometriosis is not merely a gynaecological condition but a systemic one. This systemic nature is reflected in its wide range of symptoms, which, for example, include menstrual-related symptoms such as dysmenorrhoea and dyspareunia, as well as non-menstrual symptoms affecting the bowel and bladder, and chronic pain [19]. The list of potential symptoms extends much further, with each case being unique to the woman affected. Similarly, there is a long list of comorbidities. Overall, the disease burden of endometriosis is substantial, affecting multiple aspects of women’s physical, emotional, and social well-being [20,21,22,23].

Currently, endometriosis is not curable, and treatment options are limited [24]. First-line treatment typically comprises hormonal contraception and pain medication to suppress symptoms, as well as surgical excision of endometriotic lesions when indicated [19,24]. However, lasting symptom relief is not guaranteed as lesions can regrow, and hormonal contraceptives are not suitable or well-tolerated by all women. To compound this issue, there is no certain correlation between experienced symptoms and lesion growth, impeding the predictability of the disease [25]. As a result, many women must find their own strategies to manage their unique constellation of symptoms, comorbidities and triggers. These disease management plans require a holistic and multidisciplinary approach to reduce the disease burden and improve quality of life [26,27,28,29]. Access to reliable and relevant information is therefore essential to support informed decision-making and self-management [6].

Little is known about the experiences of CALD women with endometriosis, particularly in relation to the information and support they receive. Recent evidence suggests informational support for Australian women with endometriosis, including CALD women, is sub-optimal for informed decision-making about disease management [30,31]. However, it remains unclear whether CALD women perceived this informational support differently from non-CALD Australian women. Furthermore, while the information needs of women with endometriosis have been examined internationally [32,33,34,35] and nationally [36,37], to date, no study has specifically explored the information needs of CALD women with endometriosis.

CALD communities often face challenges related to health literacy, language, and cultural barriers, all of which can limit access to information and appropriate care. Understanding CALD women’s information needs and preferences is therefore essential to ensure that information about endometriosis is accessible, relevant, and culturally appropriate.

This qualitative descriptive study aimed to explore the information needs and preferences of CALD women living with endometriosis.

## 2. Methods

This study used a qualitative descriptive design to provide an in-depth exploration of CALD women’s information needs and preferences. Qualitative descriptive studies aim to describe phenomena rather than provide explanations, offering an opportunity to gain initial insights into research areas that are currently poorly understood, without the need for a framework or theory [38]. This approach was considered appropriate given the paucity of evidence regarding the information needs and preferences of CALD women living with endometriosis. Further, this approach allows for close adherence to the data, facilitating a profound comprehension of participants’ experiences [38]. La Trobe University Human Ethics Committee granted ethical approval for the study (HEC24359).

### 2.1. Setting and Context of the Study

This study was set in the state of Victoria, which has the second-largest CALD population in Australia [39]. A state-wide context was chosen over a national study to be able to offer an in-person interview.

### 2.2. Sampling and Sample Size

Recruiting participants from CALD communities can be challenging due to factors such as communication barriers, mistrust of research, and consent processes [40]. Given these challenges, a combination of convenience and snowball sampling was used to enhance recruitment [41]. The primary aim was to achieve thematic saturation. A systematic review indicates that 9–17 interviews are required to achieve data saturation [42]. Data analysis was conducted concurrently with data collection to assess the point at which thematic data saturation was reached [43]. Recruitment ceased once no new insights relevant to the research aim emerged, indicating that thematic saturation had been reached.

### 2.3. Eligibility Criteria and Recruitment

Women were eligible for participation if they were aged over 18 years, resided in Victoria, Australia, and had either a confirmed or suspected diagnosis of endometriosis. Women were also required to be from CALD backgrounds, which was defined as having at least one of their parents born in a non-English-speaking country and/or a language other than English was primarily spoken at home. This broader definition of CALD, rather than only including first-generation migrants, was adopted as research shows that second-generation Australians continue to be influenced by their cultural background [44]. Lastly, women needed to speak sufficient English to engage in an interview.

The following outlets were used for recruitment: personal networks, social media platforms, and healthcare practitioners who were asked to circulate the study invitation. Additionally, organisations including women’s health services and networks, Primary Health Networks in Victoria, public libraries, and CALD-specific organisations such as the Ethnic Communities Council of Victoria and the Victorian Multicultural Commission were invited to share the study invite. Lastly, the Health Consumer Centre distributed the study invite through their newsletter and social media. Given the recruitment approach, no prior relationship existed between the researcher and the participating women.

### 2.4. Data Collection

Semi-structured interviews were used to elicit women’s perspectives on their information needs and preferences [38]. The interview guide was developed based on a scoping review [45] on informational support for women with endometriosis. Further, interview guides of qualitative studies on CALD communities’ information needs were referenced for the wording of the interview guide to ensure clear language that was easily understandable [13,46].

While women had the option to complete their interview in person, all opted for online interviews via Zoom. Offering women the choice of interview modality ensured that the interview setting aligned with their preferences. Online interviews provide greater anonymity [47], while also saving on travel costs and time [48]. However, they required access to suitable technology [48]. In contrast, in-person interviews were perceived as more personable as they allowed for social cues, including body language and a shared physical space [48]. All interviews were conducted by the first author, using a semi-structured interview guide with open-ended questions exploring women’s information needs and preferred information sources. The interviews were audio-recorded and transcribed via Zoom (https://www.zoom.com/, accessed on 5 March 2026). The first author reviewed the transcripts for accuracy by comparing them to the recording. Minor corrections (e.g., removal of filler words or removal of word repetitions) were made only where they did not alter meaning. One interview was excluded due to an ineligible audio recording. Women were invited to review their interview transcripts to confirm accuracy and provide any additional information or amendments. None of the women requested changes to the transcript or provided further information.

### 2.5. Data Analysis

The data were analysed by the first author using inductive thematic analysis following Braun and Clarke’s six-stage framework [49]. To ensure the quality and rigour of the analysis, the process was supervised by MG and JB, with frequent discussions at each stage of the analytic process. Coding decisions, theme development, and interpretations were reviewed collaboratively, allowing for critical reflection and refinement. This iterative approach helped to enhance the credibility and consistency of the findings. The analysis was conducted using NVivo 14 software. Familiarisation with the data occurred through the transcription process and repeated reading of the interview transcripts. Initial codes were developed using line-by-line coding and subsequently reviewed and organised into preliminary categories and then themes. Themes were then iteratively refined and reviewed to ensure internal consistency and a clear distinction between themes. The final themes were defined and named and are presented in the results section, supported by illustrative women’s quotes. Each quote includes the interview ID and the woman’s age.

### 2.6. Reflexivity Statement

The interviews were conducted and analysed by the first authora female PhD candidate who is not affected by endometriosis. While this limits experiential insight into the embodied realities of the condition, it may have reduced the potential of interpreting the women’s accounts through personal experience. Further, the first author had access to higher education and has not personally experienced barriers related to health literacy. This positioning may have influenced assumptions about access to and comprehension of health information. The use of semi-structured interviews enabled the women to prioritise their own concerns and articulate challenges that may not have been anticipated. Additionally, the interview guide helped prevent leading questions while also enabling women to focus on what was important to them.

Lastly, the first author is of mixed-ethnicity and has only recently relocated to Australia. This may have influenced how cultural differences and healthcare barriers were interpreted. Reflexive notes and ongoing discussion with the research team were used to critically examine how these positionalities shaped questioning, understanding, and theme development throughout the project.

The first author holds a Bachelor’s and Master’s degrees in health economics and has conducted multiple interview studies in the past.

## 3. Results

Out of 12 women recruited, 11 interviews were included in the analysis. Interviews lasted between 27 and 89 min. All but one woman had surgically confirmed endometriosis. The sample reflected substantial cultural diversity, with two women born in Australia and nine women born overseas, representing 10 (parental) countries of birth in total. The women’s demographics are presented in Table 1, which has been previously published [50].

Two main themes were constructed from the data: 1., Information needed, and 2., Preferences for information resources. The theme 1., Information needed, contains two sub-themes: 1.1., Disease pathogenesis, and 1.2., Disease management. The second theme, 2., Preference for information resources, also comprises two subthemes: 2.1., Preferred resource format, and 2.2., Attributes of preferred information resources, which outline the women’s preferred modes of information delivery and the characteristics these resources should ideally possess.

**Figure 1:**
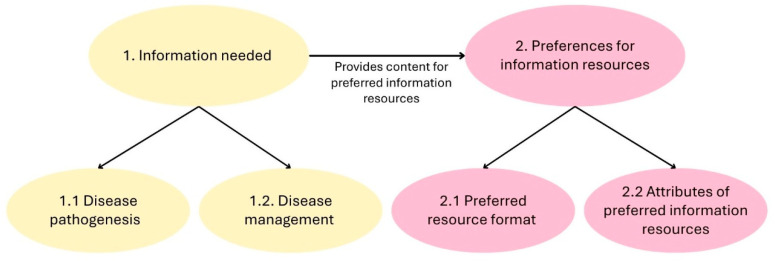
Concept map.

The themes are interconnected, as the identified information topics in the theme *Information needed* are the suggested information content of the ideal information resources described in the theme *Preferences for information resources*. A concept map of the themes and their interconnectedness is shown in Figure 1.

### 3.1. Information Needed

The theme *Information needed* describes the information needs identified by the women, including topics they had previously sought or had a current interest in. The extent and nature of their informational need varied according to symptom burden, time since diagnosis, and individual beliefs about endometriosis.

Women with milder or well-controlled symptoms reported fewer information needs, as their information seeking was usually triggered by specific symptoms. One woman explained, “I haven’t actually personally done any research as such, maybe because it’s not bothering me as much anymore. So yeah, my knowledge is quite limited” (P01; 46).

The second factor influencing information needs was time since diagnosis. While some women’s needs decreased once symptom relief was achieved, others described endometriosis as an ongoing challenge that required continuous learning. One woman shared how her understanding had evolved:

“But obviously, I’ve done a lot of research myself. I’ve had it for 24 years now, but after doing a lot of research into it, I found out a lot of various things myself which I was not aware of when I was in my teenage years or in my 20s.”

(P09; 39)

Lastly, a few women described limited information seeking due to beliefs that their symptoms would eventually stop, either following surgery or simply with time. For example, one woman initially believed that surgery would permanently resolve her symptoms and therefore stopped information seeking. However, when her symptoms returned, her information needs re-emerged:

“The surgeon said everything was removed. I thought it would help, and I think, to be honest, it probably helps. I mean, it’s just been a little over a year now, but I’m sort of back at the same cycle as I used to before, more painful and all that. So, I like, yeah, I guess that’s where I’m back at the point where I actually don’t know whether we need to start testing again or I need to look for any other supports, and again, how it fits with other things that I’m trying to navigate.”

(P10; 34)

Similarly, some women believed that their symptoms would eventually subside on their own and therefore stopped seeking information: “In the nearest future, I might not be having any of the symptoms.” (P04; 30)

While these factors influenced the scope and extent of the women’s information needs, most women identified information needs within the two sub-themes: Disease pathogenesis and disease management.

#### 3.1.1. Disease Pathogenesis

The sub-theme Disease pathogenesis describes the women’s information needs regarding the cause, symptoms, and progression of endometriosis. The women reported particularly high information needs about the cause of endometriosis. Many of the women had not heard of endometriosis before being diagnosed, leading to diverse assumptions and questions about its origin and development. For example, one woman remembered being concerned that endometriosis was a communicable disease, that she had somehow caught: “I try to ask to know if, you know, it’s an infection disease that someone transferred to me” (P06; 35). She further wondered if lifestyle factors, including nutrition, could have caused it. Even after being informed that there is no certain cause of endometriosis, she felt dissatisfied and kept wondering about the cause:

“I just had to believe that. But even up till today, most times I try to read in some articles online. Like, I go online to check some articles to know what really, really triggers endometriosis, and I wouldn’t lie, up to today I don’t think I still have the answers I am looking for.”

(P06; 35)

The lack of an explanation and the subsequent dissatisfaction with the lack of an answer often contributed to a feeling of injustice, as the women struggled to understand why they had developed endometriosis, as captured by P05 (23): “But I just felt I wasn’t supposed to have it so. Just a personal feeling I had within me. But like I don’t deserve it.”

Given the lack of an explanation, many women tried to find their own justification, highlighting the need for better informational support even in the absence of a simple answer. For example, one woman believed that emotional trauma to their mother during pregnancy can influence the child’s nervous system and trigger endometriosis: So, if your mother was nervous or scared, fearful. That could actually have an impact as to why you have endometriosis, or why I have endometriosis.” (P09; 39)

The need for a specific cause was often also linked to a hope of being able to prevent it, especially in their own daughters. For example, one woman stated, “We want to know what causes endometriosis? Is there something, I have a daughter, is there something I could do to prevent her from having endometriosis?” (P08; 30)

Overall, the need for an explanation for the cause often overshadowed any other questions and left the women confused and disappointed. Since there is currently no definitive single cause for endometriosis, it is important to provide women with information about the different potentially contributing factors and to discuss their assumptions and fears, to prevent misconceptions and misinformation.

Besides the cause, many of the women had questions about possible symptoms of endometriosis, including their own symptoms as well as the range of possible symptoms. This also included where endometriosis can occur in the body, reflecting a need for clearer information on the potential locations and manifestations of the condition:

“Like endometriosis can affect your other organs. That’s something they don’t mention to you at the start, and like the pain started like going to other places … I started getting a lot of pain, and I didn’t understand if it was a part of endometriosis, because it’s my uterus, why is it like affecting other stuff like when I go to the toilet, why am I getting pain?”

(P08; 30)

Understanding how endometriosis develops and affects the body, and therefore which symptoms can be attributed to endometriosis, was also important to the women to enable them to distinguish whether symptoms might be an indicator of another condition or their endometriosis. Not being able to distinguish endometriosis from other potential health conditions made women anxious and wonder “How do I detect that this is endometriosis?” (P03; 27)

To better understand which symptoms were caused by endometriosis, the women stressed the importance of understanding the variance in symptoms and how endometriosis presents in each individual. As one woman explained, “you could have mild endometriosis, and you could have these symptoms, or that it’s not just during your period” (P11; 29). Even though she knew about endometriosis before her diagnosis, she never considered she might have it, as she did not tick all of the textbook symptoms.

The women were interested in the progression of the disease, the “lifelong impact” (P10; 34), and wanted information and support to combat the uncertainty of the future. As one woman noted, “endometriosis can show itself during any stage of a woman’s life” (P11; 29), which confused her as she developed symptoms later in life:

“And I think like I think just for women to kind of know to keep track of symptoms throughout their cycle as they grow older, that it’s not like they could have signs of endometriosis from the onset of having their first cycle, or it could be later on down the track, like for me.”

(P11; 29)

For older women, the question of how their endometriosis would change during menopause brought many uncertainties. For example, one woman described the uncertainty she felt about how menopause might affect her endometriosis:

“How is that going to affect endometriosis? I still don’t know how that hormone change would affect the future. Is that the reason why the endometriosis tissue becomes very solid because of the menopause, and not having enough hormones? … Does it mean that I should have hormone therapy, or should I wait until I go through menopause to have that?”

(P01; 46)

Lastly, a few women wondered if “maybe it will stop” (P12; 45). This quote illustrates that the progression of endometriosis needs to be discussed to set realistic expectations, and women do not endure symptoms in the hope that it will stop of its own accord.

#### 3.1.2. Disease Management

The sub-theme Disease management describes the women’s information needs in relation to treatment options, including hormonal medication and surgery, lifestyle changes such as nutrition and physical activity, fertility treatment and mental health support services. When first diagnosed, the women were interested in the range of treatment options. For example, “What are conservative options? Can one treat it aside from a surgical [sup]pression? Those are the things I would love to know about endometriosis” (P03; 27). Regarding medication, the women wanted to know about the “side effects” (P01; 46), especially of hormonal contraception. Overall, the women were often dissatisfied with being recommended the oral contraceptive pill as treatment. For instance, one woman said:

“They all do know the side effects with the [oral contraceptive] pill and what it does to the body. My question was ‘why do they provide it and why do they recommend it.’”

(P09; 39)

Furthermore, the women expressed information needs on surgical procedures, including “complications after surgery” (P03; 27) and “why it comes back, if you remove it” (P08; 30). Only one woman was currently considering a hysterectomy and was still weighing the (dis)advantages before deciding:

“But then, when I heard that hysterectomy means I would have to go on hormonal therapy, then I thought ‘no, that was going to be even worse.’”

(P01; 46)

The women expressed a need for information on lifestyle factors, as they considered this lay within their power to influence their symptoms. Many women wanted to know how physical activity and nutrition could help with managing or even reducing endometriosis symptoms. Regarding nutrition, the women wanted to know “what I can eat” (P06; 35) and “what vitamins to take” (P09; 39). In the absence of clear guidance, some women resorted to a trial-and-error approach, experimenting with dietary changes such as going “gluten-free and also lactose-free” or increasing their intake of “iron, magnesium, [and] protein” (P09; 39).

Others tried to regulate their weight, either on the recommendation of their doctors or their families. To do so, some “increase[d] … physical activity” (P07; 31); however, for others, exercise acted as a trigger for their endometriosis, amplifying their need for informational support and guidance on how to include exercise in their life:

“But isn’t exercise good for you, like exercise is good for everything? So why is it not good for endo? And I asked my GP. And she didn’t really have an answer to that. So, one thing I’m like, if exercise is good for everything, why is it bad for Endo?”

(P08; 30)

Fertility played an important role in the lives of several of the women, generating strong information needs around the risk of infertility and available fertility treatments. These women sought clarity on topics such as “AMH [Anti-Mullerian Hormone] tests and … at home fertility tests” (P07; 31) and in vitro fertilisation (IVF): “IVF if I could go for that. But it’s expensive. It’s not for the poor masses. It’s quite expensive to do” (P06; 35). Some of the women had not yet consulted their healthcare provider but were already independently seeking information: “Even if I’m not ready yet for children, I read about it” (P05; 23).

Being well-informed about fertility options, such as IVF, was seen as essential for decision-making. However, as the women noted, financial constraints and the complexity of the process could be major barriers. One woman recalled deciding against IVF due to the impact the process would have on her body:

“My gynaecologist … asked me the question, do I want to go through IVF or egg freezing. I was thinking about it. I still wasn’t sure what to do at that point. But it was actually not until last year I really thought about it. But then, when they spoke to me about the process, I thought ‘forget it’. There’s no way I’m going through that process because they have to track your period, then they have to put injections into you. And I’m just like, Oh my God. And because the injections are putting hormones into your body. I’ve had enough of dealing with my period on a daily basis.”

(P09; 39)

For some women, access to accurate and hope-inducing fertility information had a positive impact on their emotional well-being. One woman described how reading about fertility outcomes gave her reassurance:

“Positive things I actually got from the information were that people, or definitely young ladies who actually have endometriosis, can end up being pregnant in the future—at least 70%. When I read the article, I just felt a newfound hope in me that at least all hope is not lost.”

(P05; 23)

Mental health was another key area of concern, with the women expressing a need for information on “the emotional, the mental symptoms” (P04; 30) associated with endometriosis. Many of the women described experiencing anxiety, depression, and emotional distress linked to their symptoms and the broader impact of the condition on their lives. As one woman recounted: “Just thinking about it now I’m getting anxiety … I’m like, okay, I don’t want to die right now. It was just like all these emotions hitting” (P08; 30).

The women emphasised the importance of learning how to manage their mental health and sought information on “how people with endometriosis can live well, like living well with endometriosis, how to cope with depression [and] anxiety related to endometriosis” (P05; 23). Some women also reflected on how psychological healing improved their physical symptoms:

“Not being validated, neglected as a child, being dismissed … those are the main ones. And if you’ve gone through that trauma as a child, that severely impacts you as an adult. As I’ve gone through that process now, my symptoms have got so much better with healing my past and moving past my trauma.”

(P09; 39)

Lastly, the women expressed a need for more practical guidance on where to access support and reliable information. They wanted to know more about endometriosis clinics, apps, and trustworthy research sources to help them manage their condition more effectively.

### 3.2. Preferences for Information Resources

The theme Preferences for Information Resources captures how women envisioned their ideal information resources to meet their information needs. The women discussed their preferences regarding the format and key attributes of the resources, emphasising that the content should contain the information needs described in the previous theme. Two sub-themes (preferred resource format and attributes of preferred information resources) describe the women’s preferences for endometriosis information resources.

#### 3.2.1. Preferred Resource Format

When describing their preferred resources, most of the women mentioned written information, typically online. Other suggestions included podcasts, webinars, and videos.

Podcasts and workshops were viewed as a potentially important source of information as they provide the opportunity to discuss experiences and, in case of workshops, actively ask questions:

“So, I would have a podcast and talk about it, invite women who have endometriosis to talk about it, talk about their different experiences, and whatever they go through. I would have workshops like endometriosis workshops, invite all the women to come in, ask all the questions and just talk to each other and share their experiences.”

(P08; 30)

One woman recommended that women’s health clinics could offer these workshops, which could be online to make them more accessible:

“Women’s health, one service open nearby. So that’s also very good if information [is] available for the women’s health kind of health service there. And even a workshop, again, doing an online workshop, that’s really good”.

(P12; 45)

The women suggested that a question and answer format could also be transferred to online resources using forums, where women can ask questions that will be answered by other community members or by healthcare professionals monitoring the forums: “a community page where people can put their answers, questions, and then people can answer who are living with endometriosis” (P01; 46).

“However, what was apparent was that preferences were highly individualised, with some women valuing face-to-face information, while others preferred the privacy and accessibility of online platforms. Importantly, many of the women emphasised that the format was less important than the quality of information. As one woman explained: ‘Doesn’t matter what format it is in as long as it’s accurate information and not incorrect information’”.

(P09; 39)

#### 3.2.2. Attributes of Preferred Information Resources

The women indicated that information resources needed to have certain attributes such as being up-to-date, accurate and linguistically accessible. Further case studies and information for family and friends were also seen as important by some of the women.

The woman wanted information that was up to date and reflected new research insights. As one woman explained, if she were to build an information resource, she would continually update the content as new insights became available: “And the more information I get, the more information I’ll put out there, but so far we didn’t have much, you know.” (P08; 30) Besides accuracy and currency, clarity of information was highlighted. The need for clarity was interconnected with language as the women wanted information that was easy to understand, avoiding excessive medical jargon and instead using “layperson’s language” (P07; 31). One woman stated she preferred “Websites that do not use lots of medical terminologies that will end up confusing me. Something very clear and readable” (P03; 27).

However, when asked about taboos in language, another woman with a healthcare background underscored the importance of correct medical terminology, particularly regarding genital anatomy:

“When I am with my clients, I use, or even with my own kids, I’ve always used just the medical terms for it. I’ve never just used … So, if I’m talking about vagina, it’s vagina, it’s nothing else. And when it’s penis, it’s penis.”

(P01; 46)

Less text was also preferred by most of the women since it was seen as easier to understand and process, and information resources should make use of visualisation to “draw attention” (P05; 23) and to convey information. One woman described her ideal format as a mix of text and visuals:

“So maybe pictures … and then little details next to it. What happens and then maybe like a little paragraph underneath … so it’s not so much writing, and it kind of breaks up … the picture kind of sits in my head for me.”

(P01; 46)

A few women also felt there was a need for translated resources to break down language barriers:

“Languages, definitely, the different languages, they can change their languages to the most common ones. And also some of the not so common ones, depending on the, yeah, if you’re doing it for Melbourne, then it depends where. What kind of people we have, cultural background people we have in Melbourne, and just kind of include some of the languages that are not just main Arabic, or you know, Italian and French, and Chinese and Indian, because there are a lot of other minority groups as well that also suffer from endometriosis. It’s not limited to any one culture.”

(P01; 46)

One woman noted that translations would be the only possibility to create CALD-specific information resources: “I sometimes I do wonder, though, how can you make things culturally specific? I mean, apart from whether you might translate it into a different language.” (P07; 31)

Some of the women also felt that the use of case studies or stories of other women with endometriosis lived experience could further enrich information resources and make them more appealing, by validating and reassuring women that “she is not the only one going through this” (P03; 27). Additionally, lived experience stories can help women compare symptoms and experiences.

“Examples or case studies or stories, whether they’re in a video format or in a written format, where you can read about someone who may or may not have similar symptoms and a similar experience as you.”

(P07; 31)

Further, some of the women wanted messages of encouragement and reassurance integrated into information resources. One woman wanted to let others know that their condition is not their fault, something she had at first thought before learning more about endometriosis:

“Something they don’t know is it’s not your fault. That’s 1. Second of all, nothing that you’ve done or you’re gonna do is causing you that endometriosis. So take it easy on yourself.”

(P08; 30)

Some of the women also suggested that information resources should include resources for family members and partners, to help them understand endometriosis and learn how to provide support. As one woman explained, more information needs to be conveyed to family members to reduce dismissal and stigma and to improve support for women with endometriosis from their partners and family:

“And also, maybe some information about men as well, because if they’re living with women with endometriosis, there are a lot of things that they should understand as well. Especially, you know, having sex, and if that’s painful for them, then there are ways if there is any. So, maybe a website for men to refer to—like a little link—if women are reading it and want to make people around them understand what they’re living through without having to obviously go through all the details. … So look, in a lot of cultures, oh yeah, they just dismiss [it] with things like ‘Oh, yes, women’s problem,’ or ‘it’s that time of the month,’ or something like that. Endometriosis is more than just normal menstruation and just normal pain.”

(P01; 46)

Lastly, one woman expressed her wish for better information for “all community, then this is preventable; and that’s the thing: we have to share with all the community members” (P12; 45).

## 4. Discussion

This study aimed to explore the information needs and preferences of CALD women living with endometriosis. The findings suggest the women’s primary information needs centred around the disease pathogenesis and disease management. In regard to information preferences, the findings suggest women want resources that are easily understandable, in simple English, with minimal text and more visualisations. Resources that capture other women’s experiences and the resources that enabled interaction were also important features. The findings of the study can be meaningfully interpreted through the lens of the integrated health literacy model by Sorensen et al. [12]. The model describes how health information for the individual facilitates the navigation of the healthcare system and therefore contributes to disease prevention and health promotion on a population level. Our findings have the following implications:

(1) Disease pathogenesis and disease management as the main information needs

Sorensen et al. describe how understanding health information is a key aspect in the health literacy process, which depends on perceived utility. The findings suggest the women’s primary information needs centred around the disease pathogenesis and disease management. Consistent with these findings, previous international and national research has highlighted these information topics as important to women with endometriosis more broadly, not limited to CALD populations [32,34,35,36]. For example, a German study by Zimmermann et al. [34] found that women sought information about the causes and epidemiology of endometriosis, as well as diagnostics and treatment options. However, a Finnish study [32] reported an interest in a much wider scope of information topics including causes, disease progression, treatment options, fertility, self-management, comorbidities, menopause, treatment side effects, pregnancy, and lifestyle factors, as well as communication within their social networks and access to additional resources. Similarly, a recent study highlighted Austrian women’s information needs on alternative treatment, endometriosis as a chronic condition, different life stages, specialised doctors and clinics as well as financial and legal advice [35]. This shows that while disease pathogenesis and management are important topics, not just for CALD women but for the wider endometriosis community, previous research has found an even broader scope of informational needs. In the Australian context, Cox et al. [36] surveyed women with endometriosis (CALD status not reported) and found that in-depth information about laparoscopic surgery and self-management strategies was particularly valued. While these findings overlap with the disease management needs identified in the current study, the Australian data did not emphasise the same level of interest in disease aetiology or progression. Further research is needed to better understand why information about disease causation and progression may be of particular interest to CALD women. It remains unclear whether this reflects insufficient communication regarding the scientific uncertainty surrounding the cause of endometriosis or cultural factors that shape health beliefs. It might be a possibility that the comparatively focused nature of the reported information needs reflects more fundamental informational gaps. That is, the women may have prioritised questions about cause and progression because these areas had not yet been adequately addressed, potentially limiting their ability to identify or articulate more nuanced informational needs.

(2) Written information resources should use layperson language, with minimal text and added visualisations

In alignment with the integrated health literacy model by Sorensen et al. [12], women need to be able to access, understand, appraise and apply health information to ultimately increase health-promotion. Complexity and jargon are two influential factors for the processing and appraisal of health information [12]. To improve understanding, our findings suggest that information resources need to be written in simple English without medical jargon, with minimal text and more visualisations. Previous research has shown CALD communities prefer resources with shorter text and greater use of visuals, which can support understanding and engagement [51]. The need for tailored information resources has also been supported by the Australian National Preventive Health Strategy which recommends the development of health information for CALD populations to enhance accessibility and comprehension [52].

(3) Interactive health information, lived experience accounts and resources for family and friends as helpful

Making information resources more approachable or interactive, by including accounts of lived experience and offering webinars or workshops, could further improve the uptake of health information and contribute to better healthcare usage and health promotion [12]. Lastly, our findings suggest offering health information not just for affected women but for the community. The need for information resources for the social network of women with endometriosis has been echoed in the recent literature [35].

### 4.1. Limitations and Strengths

Several limitations should be acknowledged when interpreting the study findings. Although thematic saturation was achieved, the number of participating women was relatively small. Given the heterogeneity of CALD communities, it cannot be assumed that the full range of experiences with information needs and information preferences of CALD women with endometriosis has been fully captured or represented. Nevertheless, the consistency and overlap across interviews suggest that the key themes identified are robust and credible and provide easy-to-implement recommendations for health information resources. Furthermore, only English-speaking women were eligible to participate, which excluded those facing greater language barriers when seeking information. The study findings might therefore exclude the information needs and preferences of those who are the most marginalised and who might have substantial barriers to navigating and accessing the healthcare system. It is not possible to draw strong conclusions about the needs of non-English-speaking women, since they were not included in this study. The decision to only include women who speak English was based on feasibility considerations. Similarly, women affected by stigma may not have been represented in this study, as they might be less likely to volunteer to participate in research. It is also possible that such women remain undiagnosed due to a lack of awareness or stigma preventing them from seeking medical care. This highlights the need for further research into endometriosis awareness and experiences among CALD women. Lastly, our recruitment approach may have biased the sample towards women who are more comfortable using digital technology and frequently access online resources.

There is also the potential for recall bias regarding women’s information needs and sources. Some women may not have accurately recalled all discussions with healthcare providers or the information they sought online or through peers. As a result, the findings on unmet information needs may be biased towards areas where women felt dissatisfied or lacked sufficient answers. Nonetheless, this highlights the areas with the greatest need for improvement in information provision and communication.

Lastly, it should be noted that the interviews significantly differed in duration, with the variance reflecting the time since diagnosis and individual disease burden, both of which influenced the scope of women’s information needs.

This study was the first to examine the information needs of CALD women with endometriosis in Australia and their preferences for information sources. The semi-structured interviews provided in-depth, personal insights into the experiences of women who are often underrepresented in research. Given the barriers CALD women face when accessing information, this study offers valuable evidence to help shape future information resources so that they are more inclusive and responsive to women’s needs.

### 4.2. Recommendations

Some preliminary recommendations for clinical practice and future research can be drawn; however, the previously described limitations of the study population need to be considered. The findings suggest a potential need for improved informational support, particularly regarding the pathogenesis and management of endometriosis. Information could be delivered through multiple accessible channels to meet different information preferences, although further research is required to determine which approaches are most effective across diverse CALD communities. The theme preferences for information resources provide insights into how written information might be made more accessible, specifically, through concise, easy-to-understand language and the use of visuals to aid comprehension. In addition to simple English versions, translations might improve inclusivity for those preferring to read in their native language. Notably, women who do not speak English were not included in this study, and their perspectives may differ substantially; dedicated research with non-English-speaking groups is therefore essential before firm recommendations can be made.

These written materials can complement and support patient education provided by healthcare professionals. Given that women often are not aware of endometriosis before being diagnosed, patient education is needed to inform women about the condition. This also includes discussing women’s information needs and tailoring the consultation to their specific questions. As the study revealed, many women’s information needs remain unmet, especially on topics where scientific understanding remains uncertain, such as the causes of endometriosis. Patient education might benefit from addressing these uncertainties transparently, reassuring women and helping them make sense of ongoing research gaps.

Healthcare consultations could also serve as opportunities to identify and address language barriers, clarify information obtained from other sources, and guide women towards credible and accessible resources. However, recognising the time constraints faced by healthcare providers, alternative approaches such as workshops or webinars offered through women’s health services may be valuable. These sessions could provide women with a supportive environment to discuss their questions, confirm their understanding, and receive guidance on where to find reliable information and practical support. Further research is required to assess the feasibility, acceptability, and effectiveness of such initiatives across diverse CALD groups.

Overall, more research to better understand the experiences of CALD women with endometriosis and their overall patient journey. This includes exploring general awareness of endometriosis within CALD communities, examining whether women can recognise endometriosis-related symptoms, and mapping their pathways to diagnosis, treatment, and ongoing support. A deeper understanding is also required of how socio-cultural factors may shape women’s experiences of endometriosis [53]. Once a broader understanding has been established, it would be valuable to undertake more focused research within specific CALD communities to identify unique cultural and contextual influences.

A summary of the recommendations can be seen in Table 2.

## 5. Conclusions

Our study suggests a need for improved information for CALD women with endometriosis. Information should be easy to understand, incorporate visual elements, and include lived experiences to explain causes, progression, symptoms, and disease management. Both English and translated versions might improve inclusion and break down communication barriers. Additionally, offering opportunities to ask questions, either through healthcare providers or in dedicated endometriosis workshops, may support women who find written information difficult to navigate. However, further research with CALD communities is required to substantiate and refine these recommendations.

## Figures and Tables

**Table 1:** Participant demographics [50].

ID	Age	Participants’ Country of Birth	Participants’ Parents Country of Birth		Languages Other than English Spoken at Home	Diagnosis Year
			Mother	Father		
P01	46	India	India	India	Gujarati	2023
P03	27	Somalia	Somalia	Australia	Somali	2023
P04	30	Ghana	Ghana	Ghana	Twi	2019
P05	23	South Africa	Australia	South Africa	-	2020
P06	35	Togo	Togo	Togo	Native Tongue (Not further specified)	2017
P07	31	Australia	India	India	Punjabi	-
P08	30	Jordan	Jordan	Iraq	Arabic	2024
P09	39	UK	Malaysia	Malaysia	Tamil	2017
P10	34	Nepal	Nepal	Nepal	Nepali	2024
P11	29	Australia	Australia	Lebanon	Arabic	2024
P12	45	Sri Lanka	Sri Lanka	Sri Lanka	Sinhala	2008

**Table 2:** Recommendations summary.

Development and Improvement of Information Resources	Clinical Practice	Further Research
Provide comprehensive information on management options and pathogenesis Communicate scientific uncertainty transparently, e.g., regarding causes Use plain language and avoid medical jargon Incorporate visuals to support comprehension Offer simple English versions Provide translated materials for women preferring their native language Disseminate information through multiple accessible channels	Integrate written resources into consultations to complement verbal education Explore and tailor discussions to women’s individual information needs Address language barriers during consultations Clarify information obtained from other sources Guide women towards credible and accessible resources Offer workshops or webinars, e.g., through women’s health services	Investigate the patient journey of CALD women (awareness, symptom recognition, diagnosis pathways, treatment, support) Examine socio-cultural influences on experiences and information needs Conduct focused research within specific CALD communities to identify contextual influences

## Data Availability

The original contributions presented in this study are included in the article. Further inquiries can be directed to the corresponding author.
